# Data on the characterization of (*N*-alkylsalicylaldiminato)bis(2-phenylpyridinato)iridium(III)

**DOI:** 10.1016/j.dib.2019.104300

**Published:** 2019-07-23

**Authors:** Soichiro Kawamorita, Anna Cho, Lingtao Kong, Takeshi Naota

**Affiliations:** Department of Chemistry, Graduate School of Engineering Science, Osaka University, Machikaneyama, Toyonaka, Osaka 560-8531, Japan

**Keywords:** Iridium, Phosphorescence, NMR data, X-ray analysis

## Abstract

Herein we report the synthesis, characterization data and photophysical properties of iridium(III) complexes having *N*-alkylated salicylaldimine and 2-phenylpyridine ligands. The structures of novel iridium complexes were assigned by ^1^H and ^13^C NMR, ^1^H–^1^H COSY, NOESY, HMQC, HMBC, HRMS, IR and XRD analysis. For further information, we obtained photophysical properties in solution and crystalline states.

**Table:** Specifications table

Subject area	*Chemistry*
More specific subject area	*Metal complexes, Photoluminescence,*
Type of data	*Synthesis (text),*^*1*^*H NMR (Figures),*^*13*^*C NMR (Figures),*^*1*^*H–*^*1*^*H COSY (Figures), NOESY (Figures), HMQC (Figures), HMBC (Figures), IR (text), HRMS (text), m.p. (text), X-ray (Table and Figures), UV–vis (Figures and Table), emission (Figures and Table), emission lifetime (Table)*
How data was acquired	^*1*^*H NMR,*^*13*^*C NMR,*^*1*^*H–*^*1*^*H COSY*, *NOESY*, *HMQC*, *HMBC (Varian Unity–Inova 500 spectrometer)* *IR (Bruker Equinox 55 spectrometer)* *HRMS (Bruker micrOTOF II spectrometer) m.p. (Yanagimoto melting point apparatus)* *X-ray (Rigaku XtaLAB P100 diffractometer)* *UV–vis (Jasco V650 spectrometer) emission (Jasco FP-6500 spectrometer)* *emission lifetime (Optical Building Blocks Corp. EasyLife-X)*
Data format	^*1*^*H NMR (raw),*^*13*^*C NMR (raw),*^*1*^*H–*^*1*^*H COSY (raw), NOESY (raw), HMQC (raw)*, *HMBC (raw), IR (raw), HRMS (raw), m.p. (raw), X-ray (analyzed), UV–Vis (raw), emission (raw), emission lifetime (analyzed)*
Experimental factors	*Synthesis, purification by SiO*_*2*_*chromatography and recrystallization*
Experimental features	*The characterization of the complexes was analyzed by*^*1*^*H NMR and*^*13*^*C NMR,*^*1*^*H–*^*1*^*H COSY, NOESY, HMQC*, *HMBC, FT-IR, APCI-MS, UV–vis and emission spectroscopies. The molecular structures and packings of the complexes in crystalline state were determined by XRD analysis.*
Data source location	*Toyonaka, Japan*
Data accessibility	CCDC 1875572 (**1a**), CCDC 1875573 (**1b**) and CCDC 1875574 (**1c**) (http://www.ccdc.cam.ac.uk/conts/retrieving.html, email:deposit@ccdc.cam.ac.uk.).
Related research article	S. Sprouse, K. A. King, P. J. Spellane, and R. J. Watts**,** Photophysical effects of metal-carbon σ bonds in ortho-metalated complexes of iridium(III) and rhodium(III), J. Am. Chem. Soc*.* 106 (1984) 6647–6653.

**Table:** 

**Value of the data** • Data of ^1^H NMR, ^13^C NMR, ^1^H–1H COSY, NOESY, HMQC and HMBC were given for characterization and to check purity of synthesized iridium complexes, which provide valuable resource for various research area. • Data of XRD analysis were provided for the structure of complexes and intermolecular interactions in crystalline state, which are useful information on the solid-state emission. • Photophysical data of UV–vis, emission and emission lifetime were provided for useful information on the development of phosphorescent materials.

## 1 Data

**Fig. 1:**
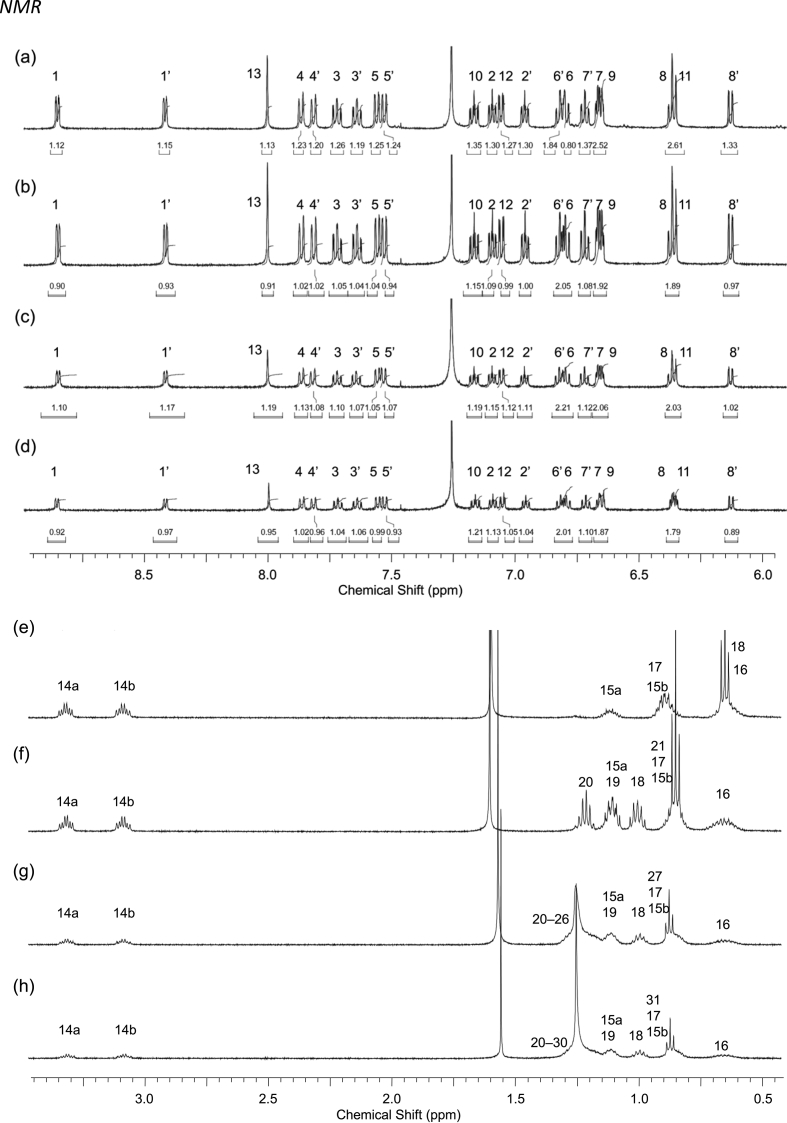
^1^H NMR spectra (500 MHz) of complexes (a, e) **1a**, (b, f) **1b**, (c, g) **1c** and (d, h) **1d**. (a–d) Aromatic and (e–h) aliphatic regions.

**Fig. 2:**
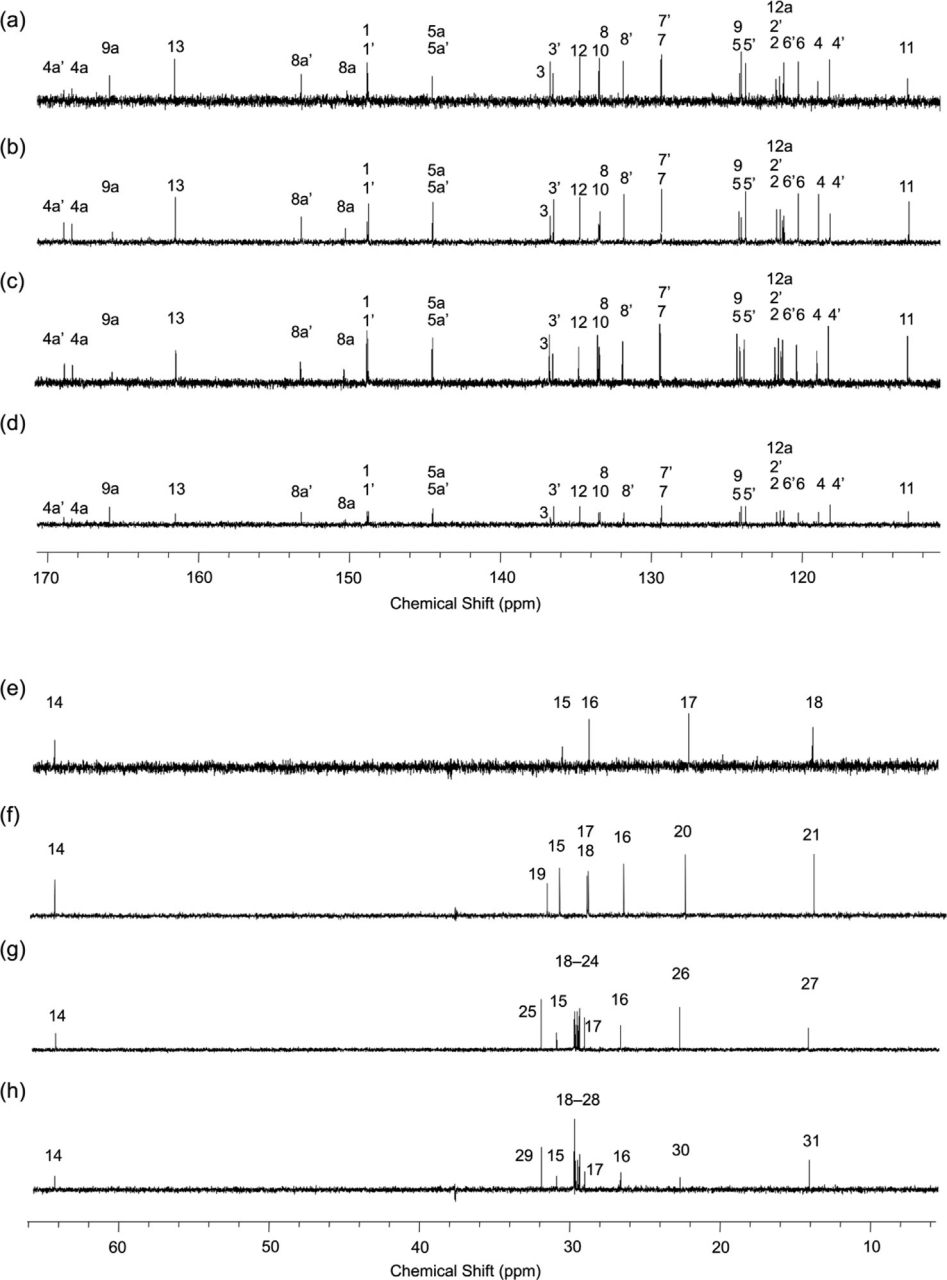
^13^C NMR spectra (125 MHz) of complexes (a, e) **1a**, (b, f) **1b**, (c, g) **1c** and (d, h) **1d**. (a–d) Aromatic and (e–h) aliphatic regions.

**Fig. 3:**
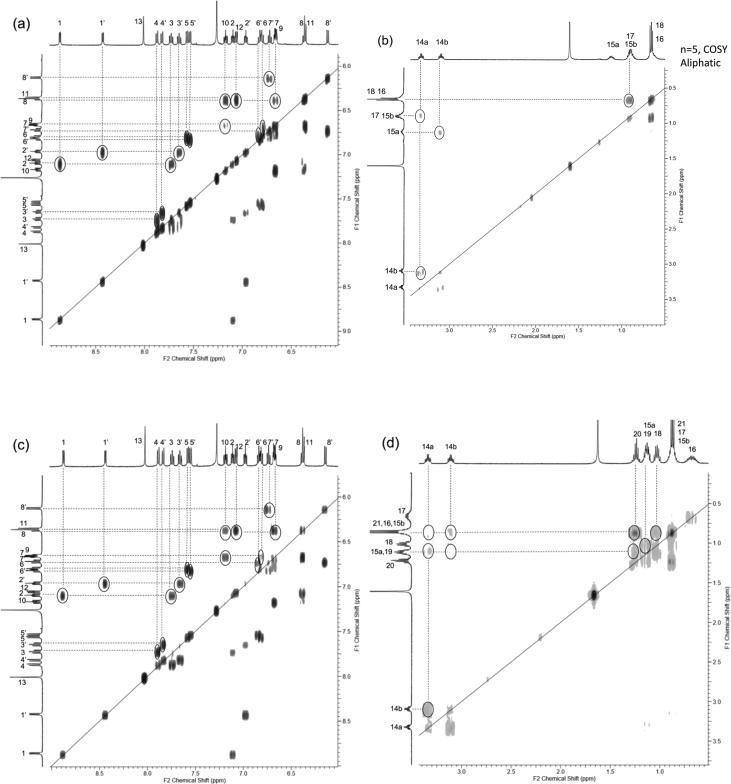

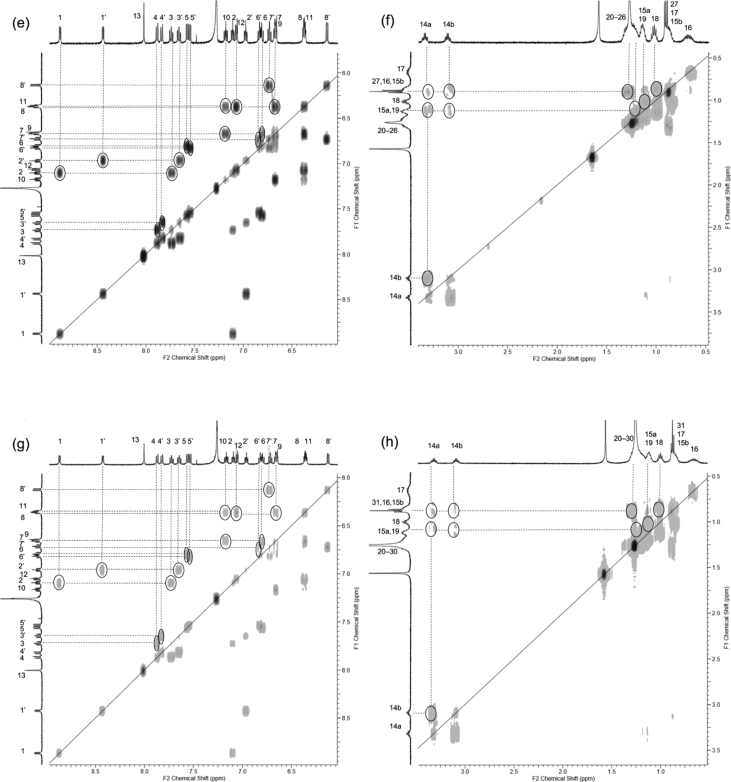
^1^H–^1^H COSY spectra (500 MHz) of complexes (a, b) **1a**, (c, d) **1b**, (e, f) **1c** and (g, h) **1d** in CDCl_3_ (298 K, number of t_1_ increments = 1024, number of t_2_ increments = 1024, number of scans = 16). (a, c, e, g) Aromatic and (b, d, f, h) aliphatic regions.

**Fig. 4:**
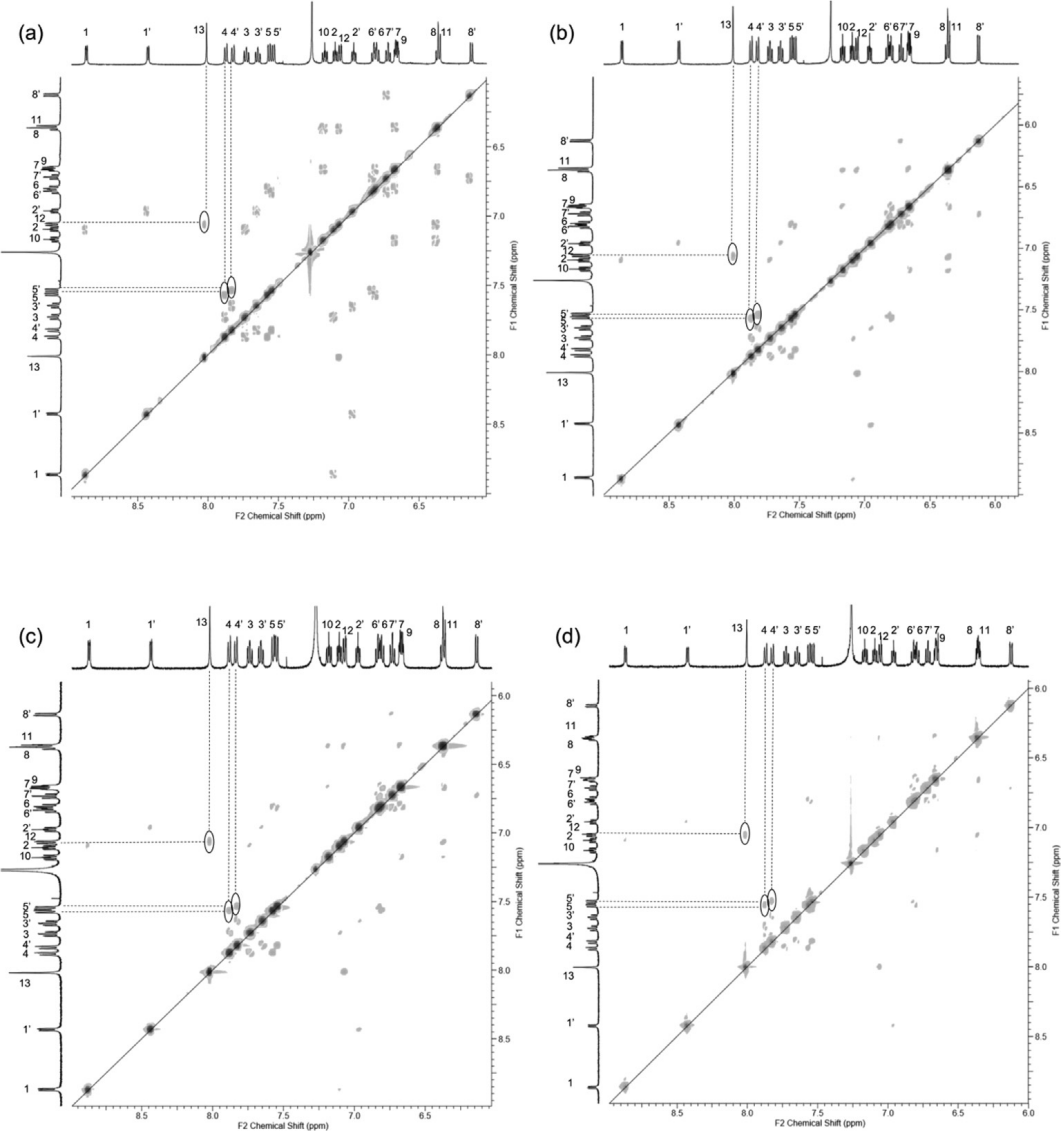
NOESY spectra (500 MHz) of complexes (a) **1a**, (b) **1b**, (c) **1c** and (d) **1d** in CDCl_3_ (298 K, mixing time = 1.00 s, number of t_1_ increments = 1024, number of t_2_ increments = 1024, number of scans = 64).

**Fig. 5:**
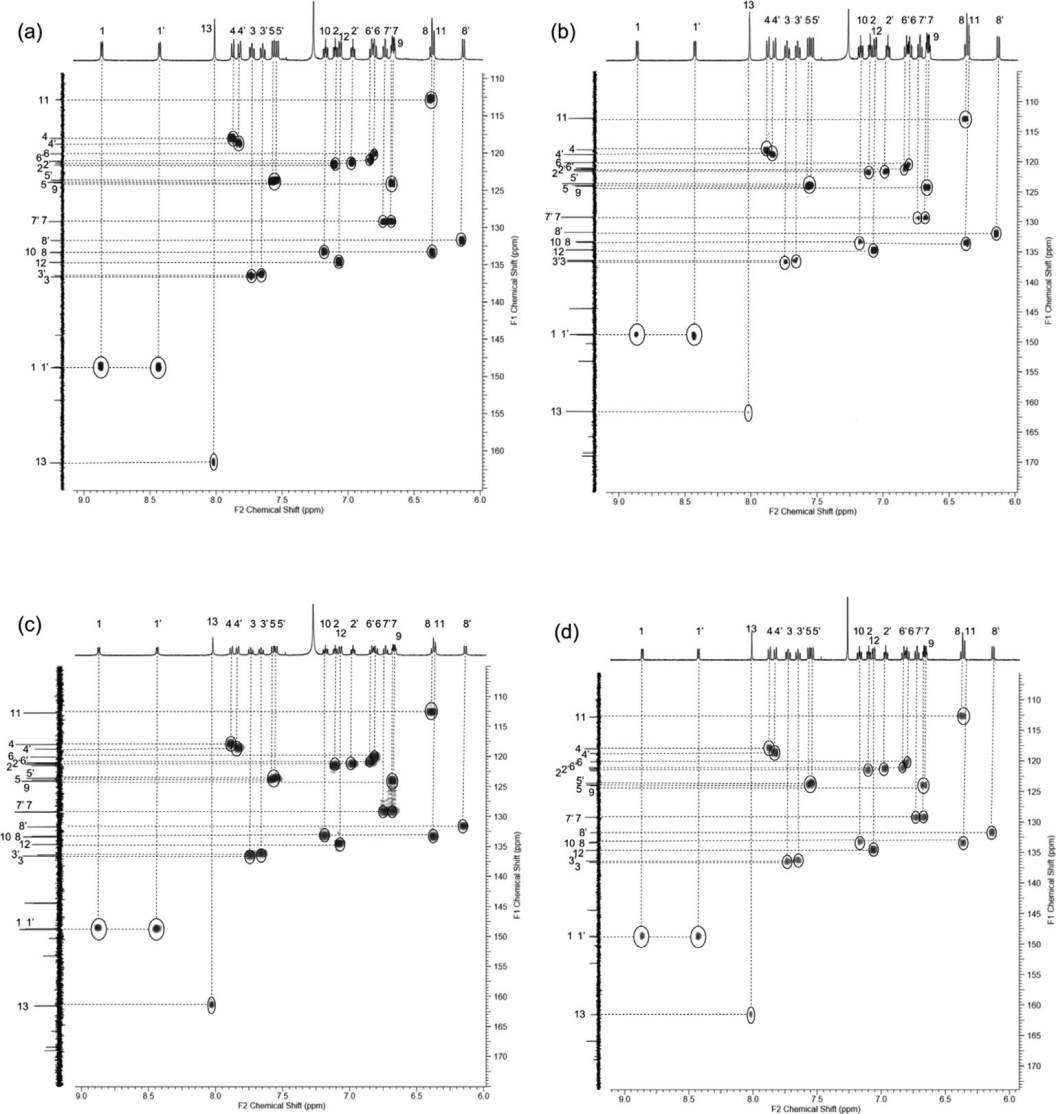
HMQC spectra (500 MHz) of complexes (a) **1a**, (b) **1b**, (c) **1c** and (d) **1d** in CDCl_3_ (298 K, number of t_1_ increments = 1024, number of t_2_ increments = 1024).

**Fig. 6:**
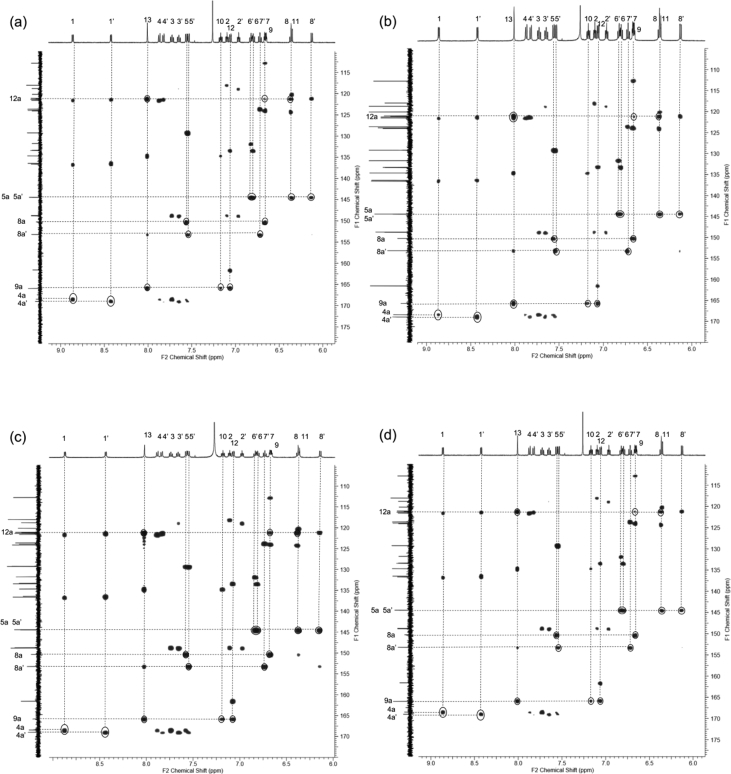
HMBC spectra (500 MHz) of complexes (a) **1a**, (b) **1b**, (c) **1c** and (d) **1d** in CDCl_3_ (298 K, number of t_1_ increments = 1024, number of t_2_ increments = 1024).

**Fig. 7:**
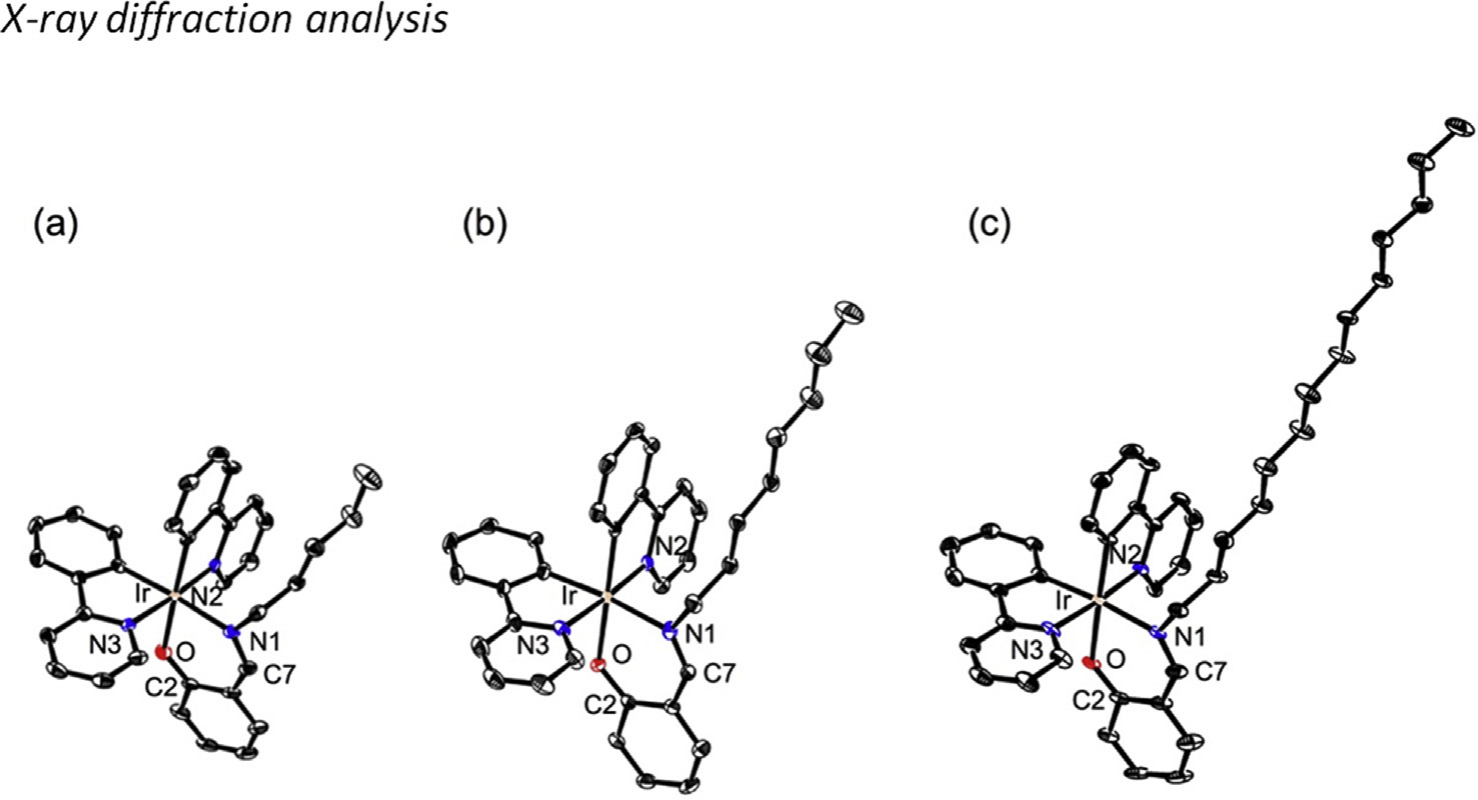
ORTEP representations of (a) Δ-**1a**, (b) Δ- **1b** and (c) Δ-**1c** as their racemic crystals. Thermal ellipsoids are shown at the 50% probability level.

**Fig. 8:**
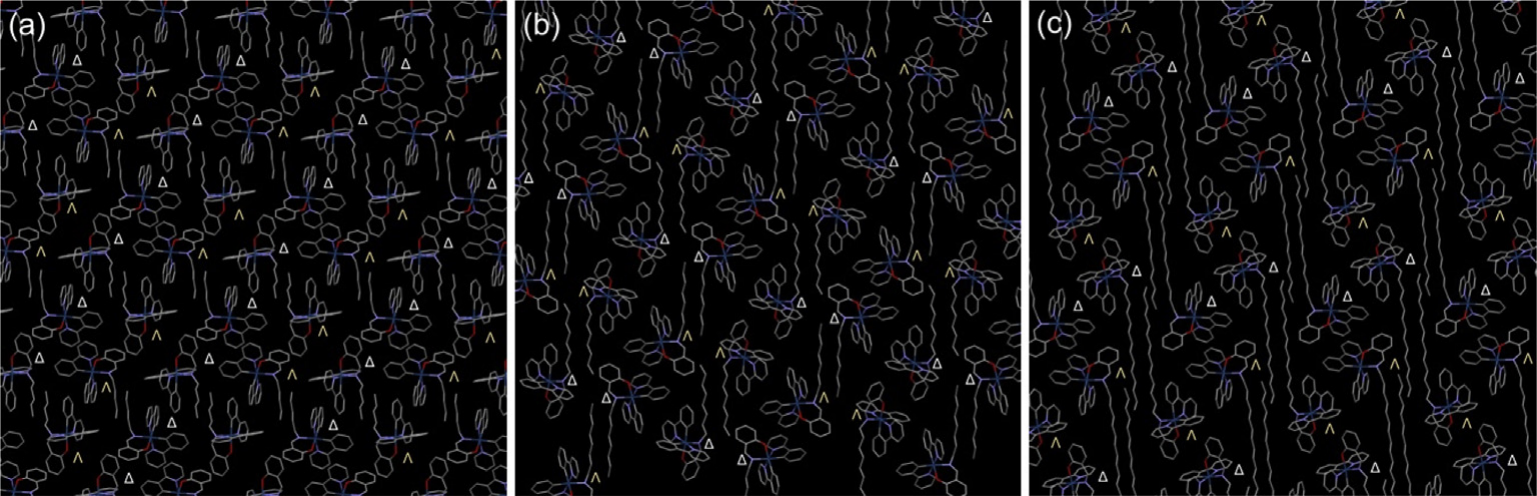
Packing in (a) **1a**, (b) **1b** and (c) **1c** crystals.

**Fig. 9:**
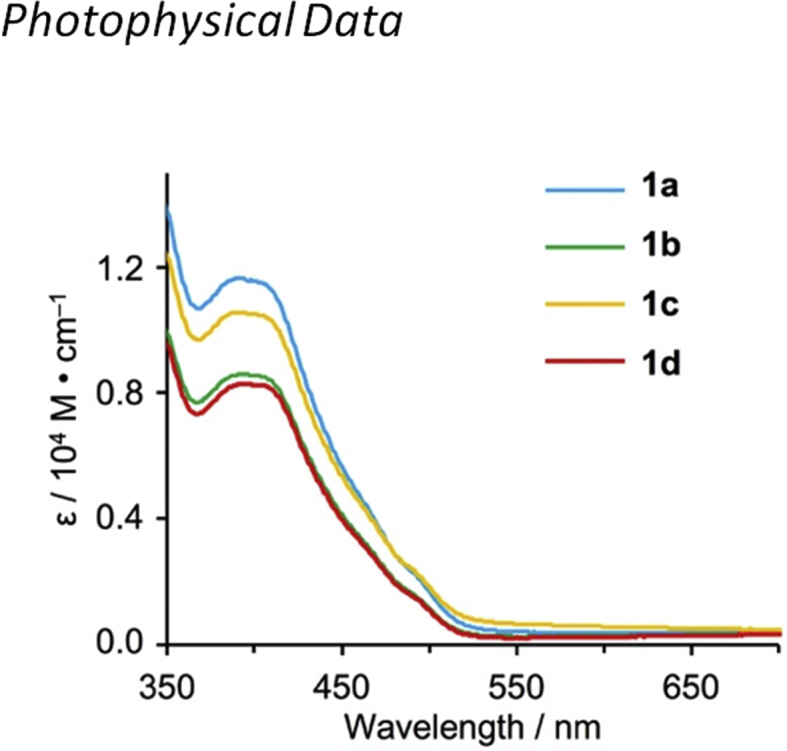
UV–vis spectra of complexes **1a**–**1d** in 2-MeTHF (2.0 × 10^−4^ M) at 298 K.

**Fig. 10:**
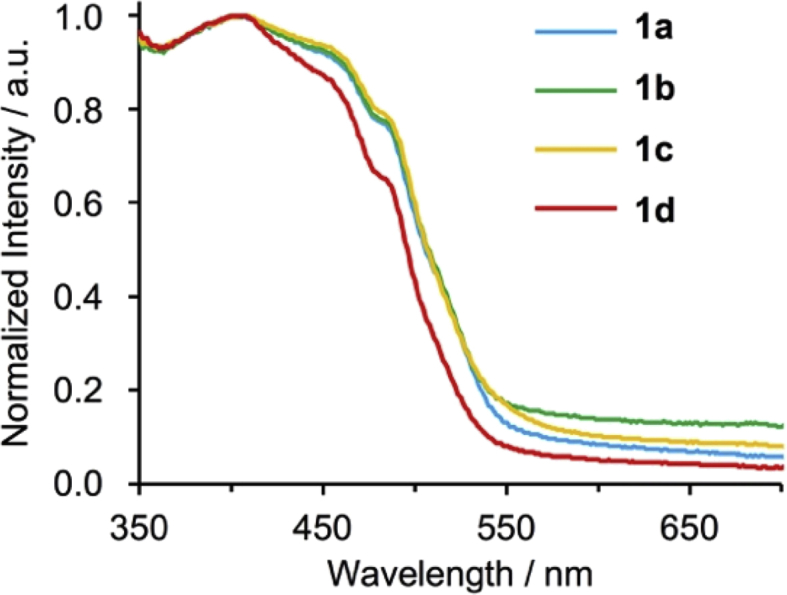
Normalized diffuse reflectance UV–vis spectra of crystals **1a**–**1d** at 298 K.

**Fig. 11:**
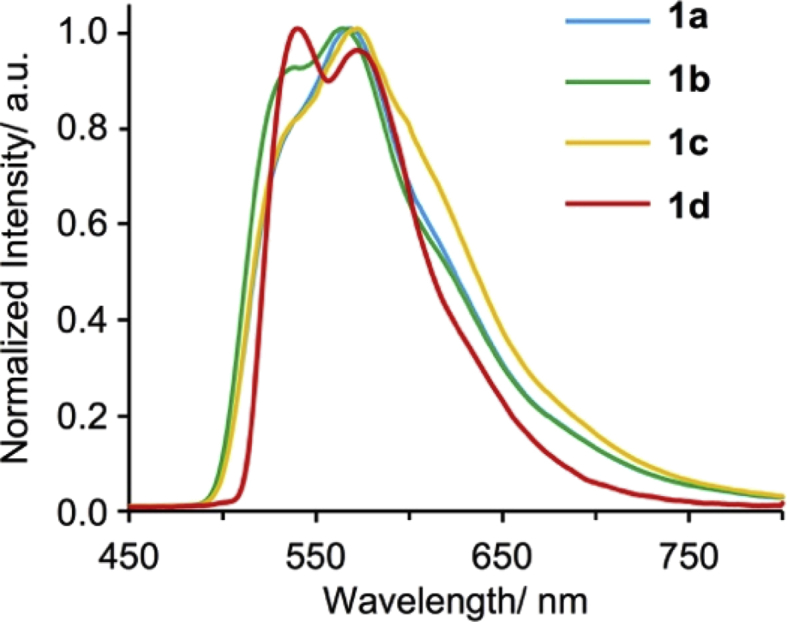
Emission spectra of complexes **1a**–**1d** in 2-MeTHF (2.0 × 10^−4^ M) at 77 K (λ_ex_ = 415 nm).

**Fig. 12:**
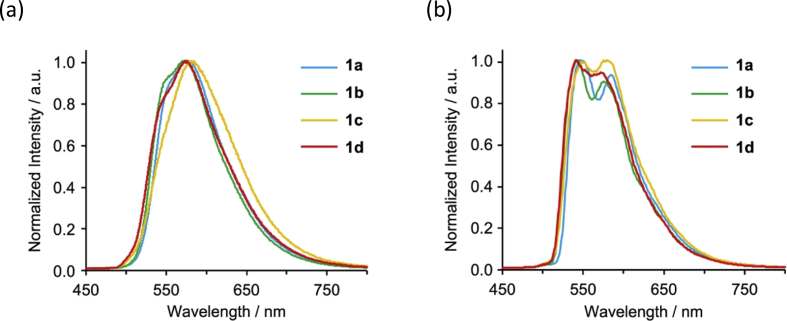
Normalized emission spectra of crystals **1a**–**1d** at (a) 298 K and (b) 77 K (*λ*_ex_ = 450 nm).

In this article we share the synthesis and characterization of iridium complexes **1a**–**1d** that exhibit phosphorescence in the solution and crystalline states. Fig. 1, Fig. 2 demonstrate ^1^H and ^13^C NMR spectra of **1a**–**1d** in CDCl_3_. Assignment of ^1^H NMR signals was made by ^1^H–^1^H COSY and NOESY experiments as shown in Fig. 3, Fig. 4. Assignment of ^13^C NMR was made by HMQC and HMBC experiments as shown in Fig. 5 and Fig. 6. Fig. 7, Fig. 8 show ORTEP drawings and packing of **1a**–**1c** determined by XRD analysis. Table 1 shows crystallographic data for **1a**–**1c.** Fig. 9, Fig. 10 show UV–vis spectra for **1a**–**1d** in 2-MeTHF and solid state. Fig. 11, Fig. 12 show emission spectra in 2-MeTHF and crystalline state. Table 2 shows photophysical data of **1a**–**1c** in 2-MeTHF and crystalline state.

**Table 1:** Crystallographic data for **1a**–**1c**.

	1a	1b	1c
formula	C_34_H_32_N_3_OIr	C_37_H_38_NO_3_Ir	C_43_H_50_N_3_OIr
*M*_r_	690.87	732.95	817.11
*T*/K	113	113	113
crystal color, habit	yellow, chip	yellow, chip	yellow, chip
crystal size/mm	1.00 × 0.50 × 0.1	0.20 × 0.20 × 0.05	0.20 × 0.05 × 0.01
crystal system	monoclinic	monoclinic	triclinic
space group	*P*2_1_/c (#14)	*P*2_1_/n (#14)	*P*-1 (#2)
*a*/Å	16.290(2)	16.334 (2)	9.6352(16)
*b*/Å	7.9.8165(11)	9.8695(13)	15.051(2)
*c*/Å	17.405(2)	19.189(3)	26.536(4)
*α*/°	90	90	75.107(5)
*β*/°	97.790(3)	94.793(4)	89.320(7)
*γ*/°	90	90	87.117(7)
*V*/Å^3^	2757.6(6)	3082.7(7)	3714.3(10)
*Z*	4	4	4
*ρ*_calcd_/g·cm^−3^	1.664	1.579	1.461
*μ* (Mo_Kα_)/cm^−1^	48.877	43.772	36.411
*F*(000)	1368.00	1464.00	1656.00
2*θ*_max_/°	55.0	55.0	55.0
No. of reflns measd	28631	35714	71214
No. of obsd reflns	6304	7043	16964
No. variables	352	379	865
*R*_1_ (*I* > 2*σ*(*I*))^a^	0.0378	0.0291	0.0485
*wR*_2_ (all reflns)^b^	0.912	0.0696	0.1166
Goodness of fit	0.975	1.008	0.9971

a *R*_1_ = Σ(|*F*_o_|–|*F*_c_|)/Σ(|*F*_o_|).

b *wR*_2_ = [Σ[w(*F*_o_^2^–*F*_c_^2^)^2^]/Σw(*F*_o_^2^)^2^]^1/2^.

**Table 2:** Photophysical data for **1a**–**1d**^[a]^.

Complex	State	*λ*_abs_ [nm]	*λ*_em_ [nm]^[c]^	*Φ*^[c,d]^	τ [μs]^[e]^	*k*_r_ × 10^−5^ [s^−1^]^[f]^	*k*_nr_ × 10^−5^ [s^−1^]^[f]^
1a	2-MeTHF^[b]^	397	–^[g]^ (561)	–^[g]^ (0.13)	(5.03)	(0.26)	(2.0)
1b	2-MeTHF^[b]^	397	–^[g]^ (527, 564)	–^[g]^ (0.13)	(4.98)	(0.26)	(2.0)
1c	2-MeTHF^[b]^	397	–^[g]^ (572)	–^[g]^ (0.15)	(5.09)	(0.29)	(2.0)
1d	2-MeTHF^[b]^	397	–^[g]^ (538, 568)	–^[g]^ (0.14)	(4.87)	(0.29)	(2.1)
1a	crystal	399	576 (550, 584)	0.02 (0.06)	(0.61)	(0.33)	(16)
1b	crystal	399	573 (541, 578)	0.03 (0.12)	(0.47)	(0.64)	(21)
1c	crystal	399	579 (553, 580)	0.02 (0.11)	(0.30)	(0.67)	(33)
1d	crystal	399	574 (541, 572)	0.03 (0.10)	(0.51)	(0.59)	(19)

^[a]^ Data were obtained at 298 K and 77 K. Values in parentheses are those measured at 77 K.

^[b]^ 2.0 × 10^−4^ M.

^[c]^ λ_ex_ = 415 nm.

^[d]^ Determined by the absolute method using an integrating sphere.

^[e]^ λ_ex_ = 415 nm.

^[f]^ Determined based on the quantum yield and lifetime.

^[g]^ No data due to non-emission properties at 298 K.

## 2 Experimental design, materials, and methods

Melting points were measured in a glass capillary using a Yanagimoto melting point apparatus. ^1^H NMR, ^13^C NMR, ^1^H–^1^H COSY, NOESY, HMQC and HMBC spectra of samples in a deuterated chloroform were recorded on a Varian Unity–Inova 500 spectrometer. ^1^H NMR and ^13^C NMR spectra were referenced to a peak of an internal TMS (0.0 ppm for ^1^H) and a deuterated chloroform (77.0 ppm for ^13^C), respectively. IR spectra was recorded on a Bruker Equinox 55 spectrometer in KBr disk at room temperature. HRMS was obtained by using a Bruker micrOTOF II spectrometer. UV–vis and emission spectra in 2-MeTHF were recorded on a Jasco V650 and a Jasco FP-6500 spectrometer respectively. Emission lifetime in 2-MeTHF was measured on an Optical Building Blocks Corp. EasyLife-X.

Crystals of **1a**–**1c** suitable for X-ray diffraction studies were prepared by recrystallization from hexane/ethyl acetate mixture, and analyzed using a Rigaku XtaLAB P200 diffractometer with Mo-Kα radiation. The structures of **1a**–**1c** were solved by direct methods and refined using the full-matrix least-squares method. In subsequent refinements, the function Σω(*F*^2^_o_–*F*^2^_c_)^2^ was minimized, where *F*_o_ and *F*_c_ are the observed and calculated structure factor amplitudes, respectively. The positions of non-hydrogen atoms were determined from difference Fourier electron-density maps and refined anisotropically. All calculations were performed with the Crystal Structure crystallographic software package, and illustrations were drawn using ORTEP [1].

## 3 Synthesis

**Figure:**
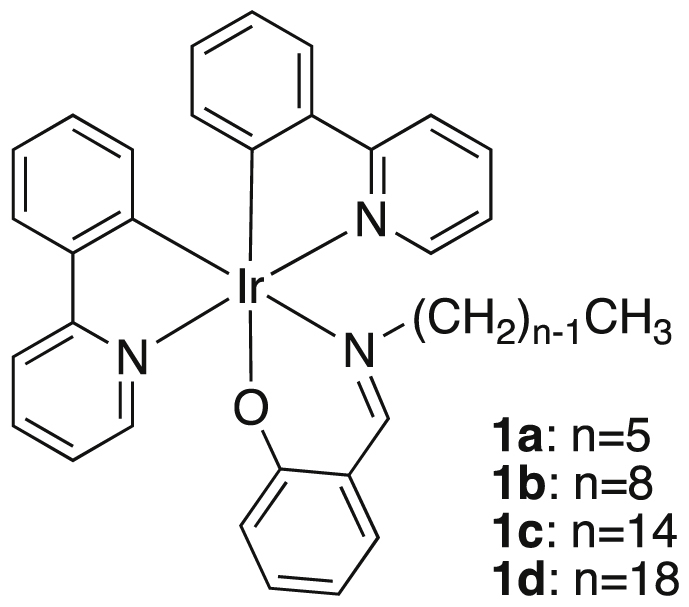

Complexes **1a**–**1d** were prepared by reaction of *μ*-chlorobis(2-phenylpyridine)iridium dimer [2] with the corresponding salicylaldimine and Na_2_CO_3_ in boiling 2-ethoxyethanol.

### 3.1 Complex 1a (n = 5)

**Figure:**
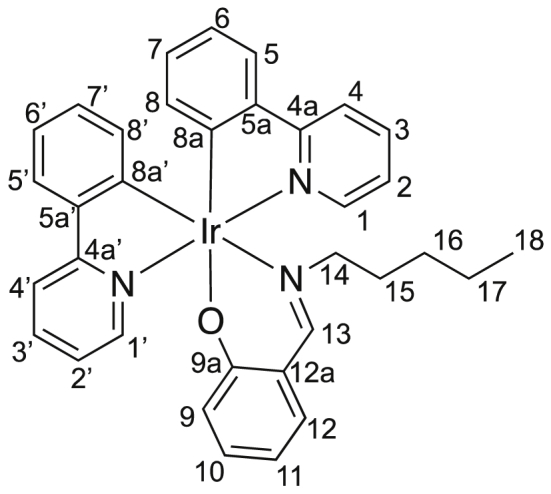

M.p. = 272–273 °C, IR (KBr): 3050, 2950, 2922, 2852, 1616, 1582, 1530, 1475, 1452, 1417, 1356, 1156, 910, 755 cm^−1^; ^1^H NMR (500 MHz, CDCl_3_) δ 0.57–0.73 (m, 5 H, *H*^*16,18*^), 0.83–0.97 (m, 3 H, *H*^*15,17*^), 1.08–1.17 (m, 1 H, *H*^*15*^), 3.10 (ddd, *J* = 5.5, 10.5, 10.5 Hz, 1 H, *H*^*14*^), 3.33 (ddd, *J* = 5.5, 10.5, 10.5 Hz, 1 H, *H*^*14*^), 6.13 (dd, *J* = 1.1, 7.5 Hz, 1 H, *H*^*8’*^), 6.36 (dd, *J* = 1.1, 7.5 Hz, 2 H, *H*^*8*^), 6.36 (ddd, *J* = 1.4, 6.9, 7.8 Hz, 1 H, *H*^*11*^), 6.66 (dd, *J* = 1.4, 8.8 Hz, 1 H, *H*^*9*^), 6.66 (ddd, *J* = 1.4, 7.5, 7.5 Hz, 1 H, *H*^*7*^), 6.72 (ddd, *J* = 1.4, 7.5, 7.5 Hz, 1 H, *H*^*7’*^), 6.80 (ddd, *J* = 1.1, 7.5, 7.8 Hz, 1 H, *H*^*6*^), 6.82 (ddd, *J* = 1.1, 7.5, 7.8 Hz, 1 H, *H*^*6’*^), 6.96 (ddd, *J* = 1.3, 5.7, 7.3 Hz, 1 H, *H*^*2’*^), 7.06 (dd, *J* = 1.8, 7.8 Hz, 1 H, *H*^*12*^), 7.10 (ddd, *J* = 1.2, 5.7, 7.3 Hz, 1 H, *H*^*2*^), 7.17 (ddd, *J* = 1.8, 6.9, 8.8 Hz, 1 H, *H*^*10*^), 7.53 (dd, *J* = 1.4, 7.8 Hz, 1 H, *H*^*5’*^), 7.57 (dd, *J* = 1.4, 7.8 Hz, 1 H, *H*^*5*^), 7.65 (ddd, *J* = 1.5, 7.3, 8.0 Hz, 1 H, *H*^*3’*^), 7.73 (ddd, *J* = 1.5, 7.3, 8.0 Hz, 1 H, *H*^*3*^), 7.82 (ddd, *J* = 0.7, 1.2, 8.0 Hz, 1 H, *H*^*4’*^), 7.87 (ddd, *J* = 0.7, 1.2, 8.0 Hz, 1 H, *H*^*4*^), 8.01 (s, 1 H, *H*^*13*^), 8.43 (ddd, *J* = 0.7, 1.5, 5.7 Hz, 1 H, *H*^*1’*^), 8.86 (ddd, *J* = 0.7, 1.5, 5.7 Hz, 1H, *H*^*1*^); ^13^C NMR (125 MHz, CDCl_3_) δ 13.9 (*C*^18^), 22.1 (*C*^17^), 28.8 (*C*^16^), 30.5 (*C*^15^), 64.3 (*C*^14^), 112.9 (*C*^11^), 118.1 (*C*^4’^), 118.8 (*C*^4^), 120.1 (*C*^6^), 121.1 (*C*^6’^), 121.2 (*C*^12a^), 121.4 (*C*^2’^), 121.6 (*C*^2^), 123.7 (*C*^5’^), 123.9 (*C*^5^), 124.0 (*C*^9^), 129.25 (*C*^7 or 7’^), 129.28 (*C*^7 or 7’^), 131.8 (*C*^8’^), 133.38 (*C*^10^), 133.41 (*C*^8^), 134.7 (*C*^12^), 136.5 (*C*^3’^), 136.7 (*C*^3^), 144.49 (*C*^5 or 5’^), 144.50 (*C*^5 or 5’^), 148.77 (*C*^1 or 1’^), 148.83 (*C*^1 or 1’^), 150.2 (*C*^8a^), 153.2 (*C*^8a’^), 161.6 (*C*^13^), 166.0 (*C*^9a^), 168.5 (*C*^4a^), 169.0 (*C*^4a’^); HRMS (APCI): *m*/*z* calcd for ^191^IrC_34_H_32_N_3_O: 689.2146; found: 689.2147 [M^+^].

### 3.2 Complex 1b (n = 8)

**Figure:**
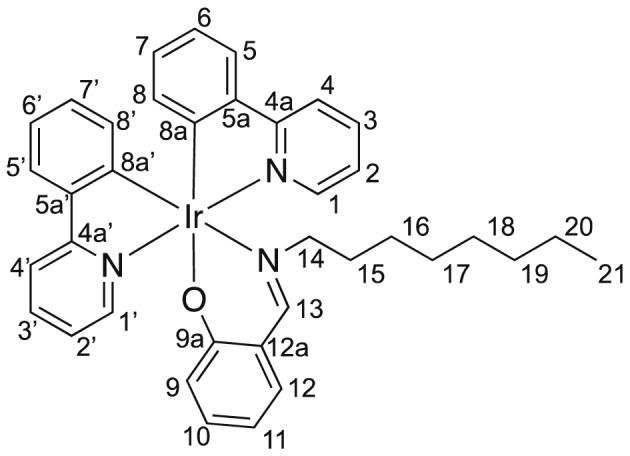

M.p. = 239–240 °C, IR (KBr): 3029, 2919, 2851, 1617, 1582, 1475, 1453, 1359, 1157, 1059, 1030, 758 cm^−1^; ^1^H NMR (500 MHz, CDCl_3_) δ 0.57–0.75 (m, 2 H, *H*^*16*^), 0.78–0.93 (m, 6 H, *H*^*15a,17,21*^), 1.01 (dddd, *J* = 7.5, 7.5, 7.5, 7.5 Hz, 2 H, *H*^*18*^), 1.08–1.14 (m, 3 H, *H*^*15b,19*^), 1.22 (dddd, *J* = 7.5, 7.5, 7.5, 7.5 Hz, 2 H, *H*^*20*^), 3.09 (ddd, *J* = 5.8, 10.1, 10.1 Hz, 1 H, *H*^*14*^), 3.33 (ddd, *J* = 5.8, 10.1, 10.1 Hz, 1 H, *H*^*14*^), 6.13 (dd, *J* = 1.1, 7.5 Hz, 1 H, *H*^*8’*^), 6.36 (dd, *J* = 1.1, 7.5 Hz, 1 H, *H*^*8*^), 6.36 (ddd, *J* = 1.4, 6.9, 7.8 Hz, 1 H, *H*^*11*^), 6.66 (dd, *J* = 1.4, 8.8 Hz, 1 H, *H*^*9*^), 6.66 (ddd, *J* = 1.4, 7.5, 7.5 Hz, 1 H, *H*^*7*^), 6.72 (ddd, *J* = 1.4, 7.5, 7.5 Hz, 1 H, *H*^*7’*^), 6.80 (ddd, *J* = 1.1, 7.5, 7.8 Hz, 1 H, *H*^*6*^), 6.82 (ddd, *J* = 1.1, 7.5, 7.8 Hz, 1 H, *H*^*6’*^), 6.96 (ddd, *J* = 1.3, 5.7, 7.3 Hz, 1 H, *H*^*2’*^), 7.06 (dd, *J* = 1.8, 7.8 Hz, 1 H, *H*^*12*^), 7.10 (ddd, *J* = 1.2, 5.7, 7.3 Hz, 1 H, *H*^*2*^), 7.17 (ddd, *J* = 1.8, 6.9, 8.8 Hz, 1 H, *H*^*10*^), 7.53 (dd, *J* = 1.4, 7.8 Hz, 1 H, *H*^*5’*^), 7.56 (dd, *J* = 1.4, 7.8 Hz, 1 H, *H*^*5*^), 7.65 (ddd, *J* = 1.5, 7.3, 8.0 Hz, 1 H, *H*^*3’*^), 7.73 (ddd, *J* = 1.5, 7.3, 8.0 Hz, 1 H, *H*^*3*^), 7.82 (ddd, *J* = 0.7, 1.2, 8.0 Hz, 1 H, *H*^*4’*^), 7.87 (ddd, *J* = 0.7, 1.2, 8.0 Hz, 1 H, *H*^*4*^), 8.01 (s, 1 H, *H*^*13*^), 8.42 (ddd, *J* = 0.7, 1.5, 5.7 Hz, 1 H, *H*^*1’*^), 8.86 (ddd, *J* = 0.7, 1.5, 5.7 Hz, 1H, *H*^*1*^); ^13^C NMR (125 MHz, CDCl_3_) δ 14.1 (*C*^21^), 22.6 (*C*^20^), 26.7 (*C*^16^), 29.0 (*C*^17 or 18^), 29.1 (*C*^17 or 18^), 30.9 (*C*^15^), 31.7 (*C*^19^), 64.3 (*C*^14^), 112.8 (*C*^11^), 118.0 (*C*^4 or 4’^), 118.1 (*C*^4 or 4’^), 120.08 (*C*^6^), 121.14 (*C*^6’^), 121.17 (*C*^12a^), 121.4 (*C*^2’^), 121.6 (*C*^2^), 123.7 (*C*^5’^), 123.9 (*C*^5^), 124.1 (*C*^9^), 129.2 (*C*^7 or 7’^), 129.3 (*C*^7 or 7’^), 131.8 (*C*^8’^), 133.3 (*C*^10^), 133.5 (*C*^8^), 134.7 (*C*^12^), 136.4 (*C*^3’^), 136.7 (*C*^3^), 144.46 (*C*^5 or 5’^), 144.49 (*C*^5 or 5’^), 148.75 (*C*^1 or 1’^), 148.81 (*C*^1 or 1’^), 150.3 (*C*^8a^), 153.2 (*C*^8a’^), 161.6 (*C*^13^), 165.8 (*C*^9a^), 168.5 (*C*^4a^), 169.0 (*C*^4a’^); HRMS (APCI): *m*/*z* calcd for ^191^IrC_37_H_38_N_3_O: 731.2615; found: 787.2611 [M^+^].

### 3.3 Complex 1c (n = 14)

**Figure:**
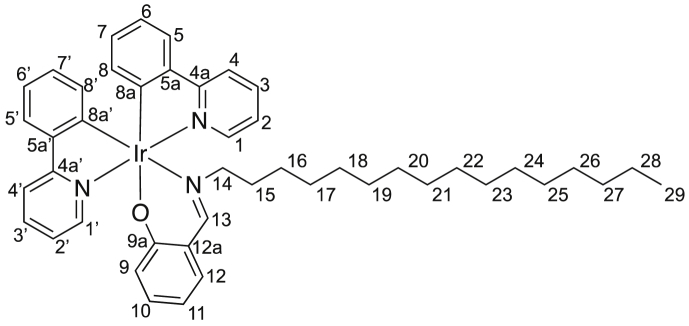

M.p. = 187.5–188.5 °C, IR (KBr): 3041, 2922, 2851, 2356, 1617, 1475, 1454, 1059. 756, 734, 675 cm^−1^; ^1^H NMR (500 MHz,CDCl_3_) δ 0.58–0.76 (m, 2 H, *H*^*16*^), 0.78–0.93 (m, 6 H, *H*^*15a,17,27*^), 1.01 (dddd, *J* = 7.5, 7.5, 7.5, 7.5 Hz, 2 H, *H*^*18*^), 1.09–1.16 (m, 3 H, *H*^*15b,19*^), 1.16–1.33 (m, 14 H, *H*^*20−26*^), 3.10 (ddd, *J* = 5.8, 10.1, 10.1 Hz, 1 H, *H*^*14*^), 3.34 (ddd, *J* = 5.8, 10.1, 10.1 Hz, 1 H, *H*^*14*^), 6.14 (dd, *J* = 1.1, 7.5 Hz, 1 H, *H*^*8’*^), 6.37 (dd, *J* = 1.1, 7.5 Hz, 1 H, *H*^*8*^), 6.38 (ddd, *J* = 1.4, 6.9, 7.8 Hz, 1 H, *H*^*11*^), 6.67 (dd, *J* = 1.4, 8.8 Hz, 1 H, *H*^*9*^), 6.67 (ddd, *J* = 1.4, 7.5, 7.5 Hz, 1 H, *H*^*7*^), 6.73 (ddd, *J* = 1.4, 7.5, 7.5 Hz, 1 H, *H*^*7’*^), 6.81 (ddd, *J* = 1.1, 7.5, 7.8 Hz, 1 H, *H*^*6*^), 6.81 (ddd, *J* = 1.1, 7.5, 7.8 Hz, 1 H, *H*^*6’*^), 6.98 (ddd, *J* = 1.3, 5.7, 7.3 Hz, 1 H, *H*^*2’*^), 7.07 (dd, *J* = 1.8, 7.8 Hz, 1 H, *H*^*12*^), 7.11 (ddd, *J* = 1.2, 5.7, 7.3 Hz, 1 H, *H*^*2*^), 7.18 (ddd, *J* = 1.8, 6.9, 8.8 Hz, 1 H, *H*^*10*^), 7.55 (dd, *J* = 1.4, 7.8 Hz, 1 H, *H*^*5’*^), 7.57 (dd, *J* = 1.4, 7.8 Hz, 1 H, *H*^*5*^), 7.66 (ddd, *J* = 1.5, 7.3, 8.0 Hz, 1 H, *H*^*3’*^), 7.74 (ddd, *J* = 1.5, 7.3, 8.0 Hz, 1 H, *H*^*3*^), 7.83 (ddd, *J* = 0.7, 1.2, 8.0 Hz, 1 H, *H*^*4’*^), 7.88 (ddd, *J* = 0.7, 1.2, 8.0 Hz, 1 H, *H*^*4*^), 8.02 (s, 1 H, *H*^*13*^), 8.43 (ddd, *J* = 0.7, 1.5, 5.7 Hz, 1 H, *H*^*1’*^), 8.87 (ddd, *J* = 0.7, 1.5, 5.7 Hz, 1H, *H*^*1*^); ^13^C NMR (125 MHz, CDCl_3_) δ 14.1 (*C*^27^), 22.7 (*C*^26^), 26.7 (*C*^16^), 29.1 (*C*^17^), 29.35, 29.42, 29.5, 29.6, 29.66, 29.71, 30.9 (*C*^15^), 31.9 (*C*^25^), 64.3 (*C*^14^), 112.9 (*C*^11^), 118.0 (*C*^4’^), 118.8 (*C*^4^), 120.2 (*C*^6^), 121.12 (*C*^6’^), 121.14 (*C*^12a^), 121.4 (*C*^2’^), 121.6 (*C*^2^), 123.7 (*C*^5’^), 123.95 (*C*^5^), 123.99 (*C*^9^), 129.2 (*C*^7 or 7’^), 129.3 (*C*^7 or 7’^), 131.8 (*C*^8’^), 133.39(*C*^10^), 133.41 (*C*^8^), 134.7 (*C*^12^), 136.5 (*C*^3’^), 136.6 (*C*^3^), 144.46 (*C*^5 or 5’^), 144.50 (*C*^5 or 5’^), 148.8 (*C*^1 or 1’^), 148.9 (*C*^1 or 1’^), 150.2 (*C*^8a^), 153.3 (*C*^8a’^), 161.6 (*C*^13^), 165.7 (*C*^9a^), 168.5 (*C*^4a^), 169.0 (*C*^4a’^); HRMS (APCI): *m*/*z* calcd for ^191^IrC_43_H_50_N_3_O: 815.3554; found: 815.3545 [M^+^].

### 3.4 Complex 1d (n = 18)

**Figure:**
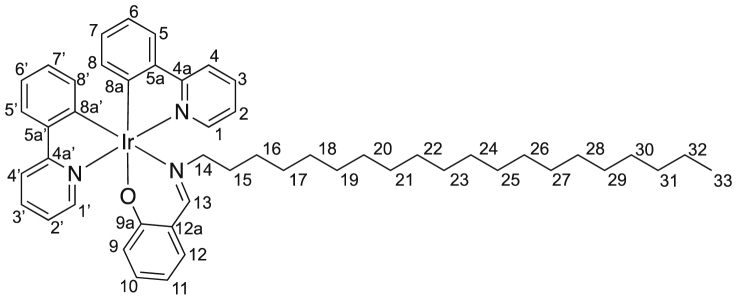

M.p. = 176–177 °C, IR (KBr): 3048, 2923, 2851, 1617, 1583, 1532, 1475, 1453, 1416, 1357, 1332, 1157 cm^−1^; ^1^H NMR (500 MHz, CDCl_3_) δ 0.58–0.75 (m, 2 H, *H*^*16*^), 0.79–0.93 (m, 6 H, *H*^*15a,17,31*^), 1.01 (dddd, *J* = 7.5, 7.5, 7.5, 7.5 Hz, 2 H, *H*^*18*^), 1.08–1.15 (m, 3 H, *H*^*15b,19*^), 1.15–1.33 (m, 22 H, *H*^*20−30*^), 3.10 (ddd, *J* = 5.8, 10.1, 10.1 Hz, 1 H, *H*^*14*^), 3.34 (ddd, *J* = 5.8, 10.1, 10.1 Hz, 1 H, *H*^*14*^), 6.14 (dd, *J* = 1.1, 7.5 Hz, 1 H, *H*^*8’*^), 6.37 (dd, *J* = 1.1, 7.5 Hz, 1 H, *H*^*8*^), 6.38 (ddd, *J* = 1.4, 6.9, 7.8 Hz, 1 H, *H*^*11*^), 6.67 (dd, *J* = 1.4, 8.8 Hz, 1 H, *H*^*9*^), 6.67 (ddd, *J* = 1.4, 7.5, 7.5 Hz, 1 H, *H*^*7*^), 6.73 (ddd, *J* = 1.4, 7.5, 7.5 Hz, 1 H, *H*^*7’*^), 6.81 (ddd, *J* = 1.1, 7.5, 7.8 Hz, 1 H, *H*^*6*^), 6.81 (ddd, *J* = 1.1, 7.5, 7.8 Hz, 1 H, *H*^*6’*^), 6.98 (ddd, *J* = 1.3, 5.7, 7.3 Hz, 1 H, *H*^*2’*^), 7.07 (dd, *J* = 1.8, 7.8 Hz, 1 H, *H*^*12*^), 7.11 (ddd, *J* = 1.2, 5.7, 7.3 Hz, 1 H, *H*^*2*^), 7.18 (ddd, *J* = 1.8, 6.9, 8.8 Hz, 1 H, *H*^*10*^), 7.55 (dd, *J* = 1.4, 7.8 Hz, 1 H, *H*^*5’*^), 7.57 (dd, *J* = 1.4, 7.8 Hz, 1 H, *H*^*5*^), 7.66 (ddd, *J* = 1.5, 7.3, 8.0 Hz, 1 H, *H*^*3’*^), 7.74 (ddd, *J* = 1.5, 7.3, 8.0 Hz, 1 H, *H*^*3*^), 7.83 (ddd, *J* = 0.7, 1.2, 8.0 Hz, 1 H, *H*^*4’*^), 7.88 (ddd, *J* = 0.7, 1.2, 8.0 Hz, 1 H, *H*^*4*^), 8.02 (s, 1 H, *H*^*13*^), 8.43 (ddd, *J* = 0.7, 1.5, 5.7 Hz, 1 H, *H*^*1’*^), 8.87 (ddd, *J* = 0.7, 1.5, 5.7 Hz, 1H, *H*^*1*^); ^13^C NMR (125 MHz, CDCl_3_) δ 14.1 (*C*^31^), 22.7 (*C*^30^), 26.7 (*C*^16^), 29.0 (*C*^17^), 29.35, 29.42, 29.5, 29.61, 29.65, 29.67, 29.71, 30.9 (*C*^15^), 31.9 (*C*^29^), 64.3 (*C*^14^), 112.8 (*C*^11^), 118.0 (*C*^4’^), 118.8 (*C*^4^), 120.2 (*C*^6^), 121.1 (*C*^6’^), 121.2 (*C*^12a^), 121.4 (*C*^2’^), 121.6 (*C*^2^), 123.7 (*C*^5’^), 123.9 (*C*^5^), 124.1 (*C*^9^), 129.25 (*C*^7 or 7’^), 129.27 (*C*^7 or 7’^), 131.8 (*C*^8’^), 133.3 (*C*^10^), 133.4 (*C*^8^), 134.7 (*C*^12^), 136.4 (*C*^3’^), 136.6 (*C*^3^), 144.46 (*C*^5 or 5’^), 144.49 (*C*^5 or 5’^), 148.76 (*C*^1 or 1’^), 148.83 (*C*^1 or 1’^), 150.3 (*C*^8a^), 153.2 (*C*^8a’^), 161.6 (*C*^13^), 166.0 (*C*^9a^), 168.5 (*C*^4a^), 169.0 (*C*^4a’^); HRMS (APCI): *m*/*z* calcd for ^191^IrC_47_H_58_N_3_O: 871.4180; found: 871.4174 [M^+^]. Anal. Calcd for IrC_47_H_58_N_3_O: C, 64.65; H, 6.70; N, 4.81. Found: C, 64.46, H, 6.61, N, 4.76.
